# Effects of Apigenin on Pharmacokinetics of Dasatinib and Probable Interaction Mechanism

**DOI:** 10.3390/molecules28041602

**Published:** 2023-02-07

**Authors:** Mohammad Raish, Ajaz Ahmad, Mudassar Shahid, Yousef A. Bin Jardan, Abdul Ahad, Mohd Abul Kalam, Mushtaq Ahmad Ansari, Muzaffar Iqbal, Naushad Ali, Khalid M. Alkharfy, Fahad I. Al-Jenoobi

**Affiliations:** 1 Department of Pharmaceutics, College of Pharmacy, King Saud University, Riyadh 11451, Saudi Arabia; 2 Department of Clinical Pharmacy, College of Pharmacy, King Saud University, Riyadh 11451, Saudi Arabia; 3 Department of Pharmacology and Toxicology, College of Pharmacy, King Saud University, Riyadh 11451, Saudi Arabia; 4 Department of Pharmaceutical Chemistry, College of Pharmacy, King Saud University, Riyadh 11451, Saudi Arabia; 5 Quality Assurance Unit, College of Pharmacy, King Saud University, Riyadh 11451, Saudi Arabia

**Keywords:** dasatinib, sinapic acid, herb drug interactions, PgP/MDR1, BCPR/ABCG2, CYP3A2, pharmacokinetics

## Abstract

Dasatinib (DAS), a narrow-therapeutic index drug, Bcr-Abl, and Src family kinases multitarget inhibitor have been approved for chronic myelogenous leukemia (CML) and Ph-positive acute lymphocytic leukemia (Ph+ ALL). Apigenin (APG) has a long history of human usage in food, herbs, health supplements, and traditional medicine, and it poses low risk of damage. The concomitant use of APG containing herbs/foods and traditional medicine may alter the pharmacokinetics of DAS, that probably lead to possible herb–drug interactions. The pharmacokinetic interaction of APG pretreatment with DAS in rat plasma following single and co-oral dosing was successfully deliberated using the UPLC–MS/MS method. The in vivo pharmacokinetics and protein expression of CYP3A2, Pgp-MDR1, and BCPR/ABCG2 demonstrate that APG pretreatment has potential to drastically changed the DAS pharmacokinetics where escalation in the Cmax, AUC_(0–t)_, AUMC_(0-inf_obs)_, T_1/2_, Tmax, and MRT and reduction in K*el*, Vd, and Cl significantly in rats pretreated with APG 40 mg/kg, thus escalating systemic bioavailability and increasing the rate of absorption via modulation of CYP3A2, Pgp-MDR1, and BCPR/ABCG2 protein expression. Therefore, the concomitant consumption of APG containing food or traditional herb with DAS may cause serious life-threatening drug interactions and more systematic clinical study on herb–drug interactions is required, as well as adequate regulation in herbal safety and efficacy.

## 1. Introduction

**Figure 1:**
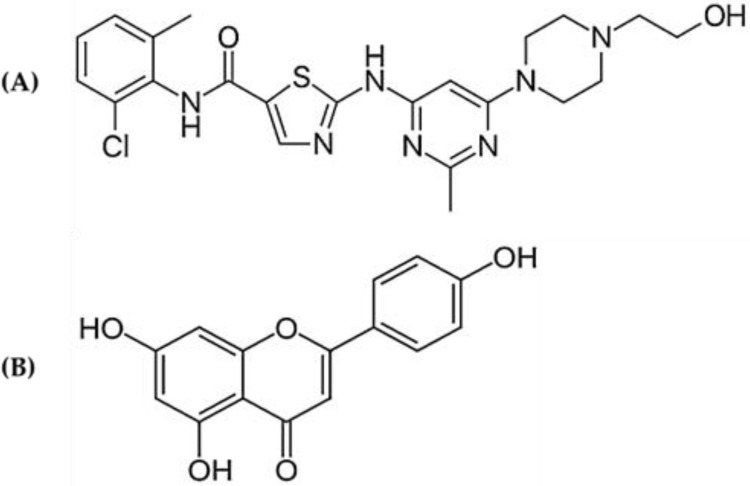
Chemical structure of (**A**) dasatinib and (**B**) apigenin.

Traditional, time-limited, nonspecific cytotoxic chemotherapy regimens are being replaced by continuous oral treatment with particular protein-targeted medicines for a number of cancers [1]. Tyrosine kinases are vital for regulating the cell cycle, cell division, apoptosis, programmed cell death, growth, and metabolism [2]. The overexpression or somatic mutations of tyrosine kinases are dysregulated in various malignancies [3]. The oral Tyrosine Kinase Inhibitor (TKI) is well known for target cancer therapy for various malignancies and has lower toxicities than conventional chemotherapies. TKIs are commonly prescribed for the treatment in patients with leukemia for prolonged periods. They are therefore commonly given together with conventional therapies may cause harmful interactions [4]. DAS is a narrow therapeutic index drug and a novel tyrosine kinase multitarget inhibitor of the BCR-ABL and Src family of kinases was permitted for the treatment of chronic myelogenous leukemia (CML) by the US FDA, as well as for Ph^+^ ALL patients who could not tolerate first-line therapy [5,6]. DAS (Figure 1A) is extensively metabolized in the liver and intestine by the CYP3A4 [7]. Additionally, DAS is a substrate of CYP3A, P-glycoprotein (Pgp; ABCB1) and Breast Cancer Resistance Protein (BCRP; ABCG2) [8,9,10]. Previous studies have stated that DAS enhances the exposure of CYP3A4 substrates simvastatin, cyclosporine, posaconazole, and midazolam, in humans and cyclosporine in rats [9,10,11,12]. DAS is associated with life-threatening toxicities such as QT prolongation, hypertension, heart failure, peripheral arterial occlusive disease (PAOD), thrombosis, myelosuppression, cytopenias, hyperlipidemia, pneumonitis, hypocalcemia, pleural effusion, and pulmonary hypertension [13].

Apigenin (APG) (4′,5,7-trihydroxyflavone) (Figure 1B) is a bioactive plant flavone present in wide variety of plants, such as celery, parsley, chamomile, and widely distributed in common food [14,15]. 4′,5,7-trihydroxyflavone (Apigenin) derivative of phenylpropanoid unit C6-C3 with glycosylated tricyclic core attached to sugar moieties through hydroxyl groups (O-glycosides) or carbon (C-glycosides). The known APG glycosides are apigenin-6-C-glucoside (isovitexin), apigenin-7-O-glucoside, pigenin 6-C-glucoside 8-C-arabinoside (schaftoside), apigenin-8-C-glucoside (vitexin), apigenin 7-O-neohesperidoside (rhoifolin), and apiin [16]. APG is extensively used in food and traditional and modern herbal medicine owing to its therapeutic activity such as antioxidant, anti-inflammatory, hepatoprotective, antithrombotic, chemo-preventive, cardioprotective, antihypertensive, anticancer, antihyperlipidemic, antidepressant, and estrogenic effects [17]. The widespread use of APG in food, herbs, health supplement, and in traditional medicine has a long human consumption history and poses little or no potential risk [18]. APG is a potent inhibitor of the cytochrome P450 (CYP) and drug transporters P-glycoprotein (Pgp) and Breast Cancer Resistance Protein (BCRP) that play a central role in several drugs’ metabolism. Therefore, APG has ability to change the pharmacokinetic profile of several drugs absorption, distribution, metabolism and elimination (ADME) via the regulatory actions of the efflux pumps Pgp and BCPR and cytochrome P450 enzymes. Dasatinib is a substrate of CYP3A, Pgp and BCRP and is subject to induction [19,20,21,22]. The concomitant use of APG may give rise to potential DAS−drug/herb interactions (DHIs). Liu et al., 2013 previously reported the pharmacokinetic interaction of apigenin with imatinib and N-desmethyl imatinib in rats [23]. The concomitant administration of substrates, metabolic inhibitors CYP3A/2C11, and drug transporters (Pgp/BCPR) inhibitors would change DAS pharmacokinetics by increasing bioavailability and organ uptake, leading to DAS−drug/herb interactions (DHIs) and toxicities. Subsequently, the identification and comprehension of these APG–DAS interactions remain important keys for risk assessment. There are no reports available on detailed mechanistic approach regarding the implication of drug metabolism (CYP3A and CYP2C11) and role of drug transporters (Pgp and BRCP) to resolve the possible interaction of APG with DAS. This study has been designed to explore the effect of APG on pharmacokinetics of DAS in rats and to resolve the possible interaction mechanism. This study will further help to aid physicians and pharmacists in the clinical management of drug–herb interactions. Careful consideration of DHI will help us create a treatment plan that is both effective and safe for the patient.

## 2. Results

### 2.1. Pharmacokinetic Interaction

**Figure 2:**
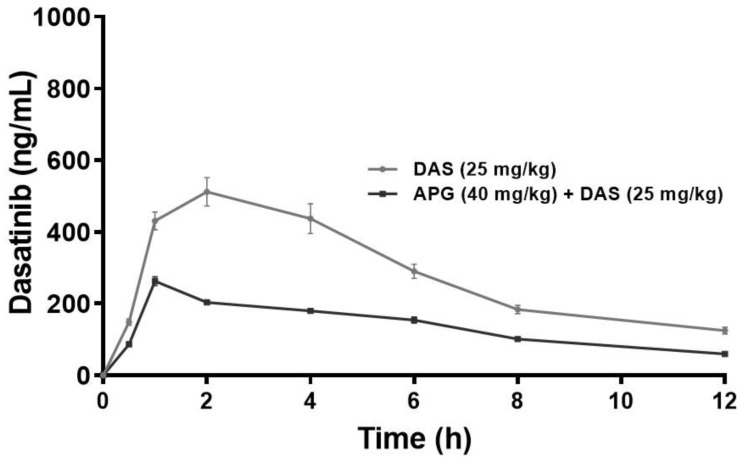
Plasma concentration–time curve of DAS and DAS along with APG (40 mg/kg p.o. for 7 days) following an oral administration in rats (*p* < 0.05).

The plasma concentration versus time profiles of the oral DAS 25 mg/kg alone and in combination with APG (40 mg/kg) pretreatment for seven days are represented in Figure 2. Pharmacokinetic parameters of DAS 25 mg/kg alone and with APG 40 mg/kg pretreatment for seven days are presented in Table 1. The pretreatment of oral APG 40 mg/kg and parallel use of DAS 25 mg/kg exhibits pharmacokinetic interaction. The DAS 25 mg/kg oral administration after APG 40 mg/kg seven days pretreatment significantly increased the plasma concentration of DAS as indicated by increased *C*_max_ (94.55%, *p* < 0.05), AUC_(0–t)_ (80.20%, *p*  < 0.05), AUMC_(0-inf_obs)_ (143.65%, *p* < 0.05), T_1/2_ (32.16%, *p* < 0.05), T max (50%, *p* < 0.05), and MRT (21.89%, *p* < 0.05) and decreased in K*el* (24.82%, *p* < 0.05), Vd (33.68%, *p* < 0.05), and Cl (49.95%, *p* < 0.05), respectively, as compared to DAS alone indicate towards increased in systemic bioavailability of DAS in plasma may be due to inhibition of CYP3 A2, Pgp/MDR1, and BCRP/ABCG2-mediated metabolism in liver and intestine, thus increasing the rate of absorption and delay in excretion of DAS.

### 2.2. Effect of Apigenin on Hepatic and Intestinal CYP3A2 Protein Expression

**Figure 3:**
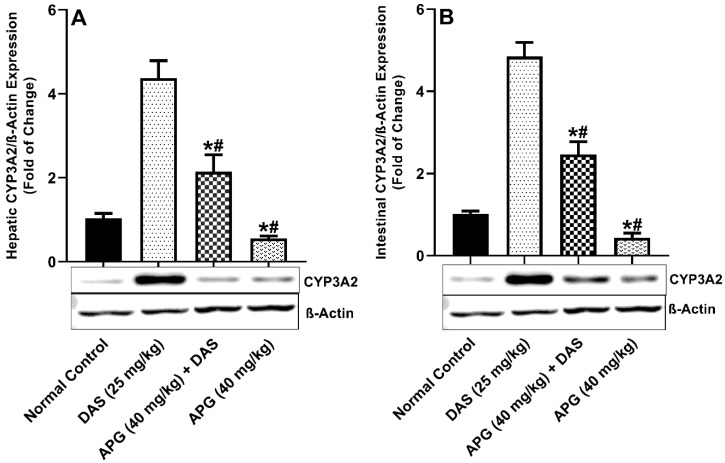
Hepatic (**A**) and intestinal (**B**) CYP3A2 protein expression in rats after DAS administration with or without APG pretreatment. All results are presented as the average ± SD. * *p* < 0.05 (Normal control); # *p* < 0.05 (DAS).

As demonstrated in Figure 3A,B, the hepatic and intestinal CYP3A2 (*p* < 0.05) protein expression of the CYP3A2 was significantly increased 76.47%-fold and 79.13% in DAS administered rats as compared with normal control rats. Chronic administration of APG (40 mg/kg b.w.) pretreatment for seven days to DAS-administered rats significantly inhibits 50.92% and 49.22% hepatic and intestinal CYP3A2 protein expression as compared to DAS administered rats. APG (40 mg/kg) only pretreatment led to the inhibition of 87.24% and 90.97% of hepatic and intestinal CYP3A2 protein expression as compared to normal rats.

### 2.3. Effect of Apigenin on Hepatic and Intestinal Pgp/MDR1 Protein Expression

**Figure 4:**
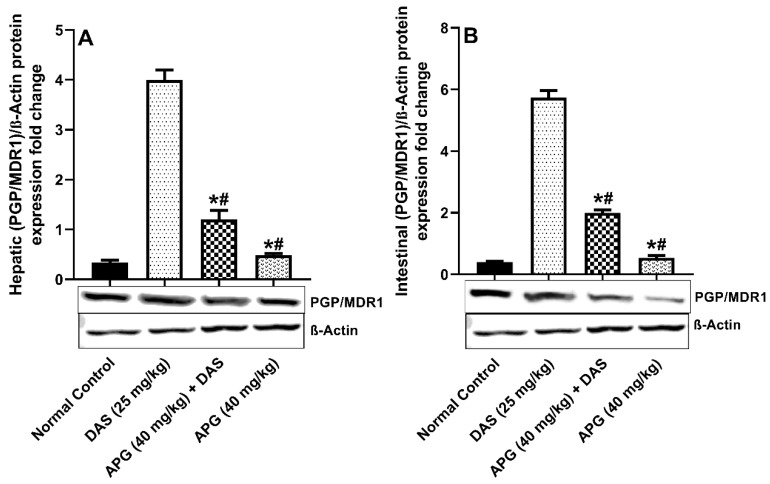
Hepatic (**A**) and intestinal (**B**) PGP/MDR1 protein expression in rats after DAS administration with or without APG pretreatment. All results are presented as the average ± SD. * *p* < 0.05 (Normal control); # *p* < 0.05 (DAS).

The inhibitory potential of APG on Pgp/MDR1 protein expression was investigated and shown in Figure 4A,B. hepatic and intestinal PGP/MDR1 protein expression was significantly decreased after APG pretreatment compared to that in normal rats. DAS peroral administration caused significant induction of (93.08% and 91.65%, respectively) (*p* < 0.05) hepatic and intestinal protein expression of Pgp/MDR1 compared to that in normal rats. The APG-pretreated rats showed significant inhibition of the Pgp/MDR1 protein, that is 65.38% and 69.90% inhibition, respectively, as compared to DAS alone (*p* < 0.05). APG (40 mg/kg) only pretreatment led to the inhibition of 90.67% and 87.84% of hepatic and intestinal Pgp/MDR1 protein expression as compared to normal rats.

### 2.4. Effect of APG on Hepatic and Intestinal BCRP/ABCG2 Protein Expression

**Figure 5:**
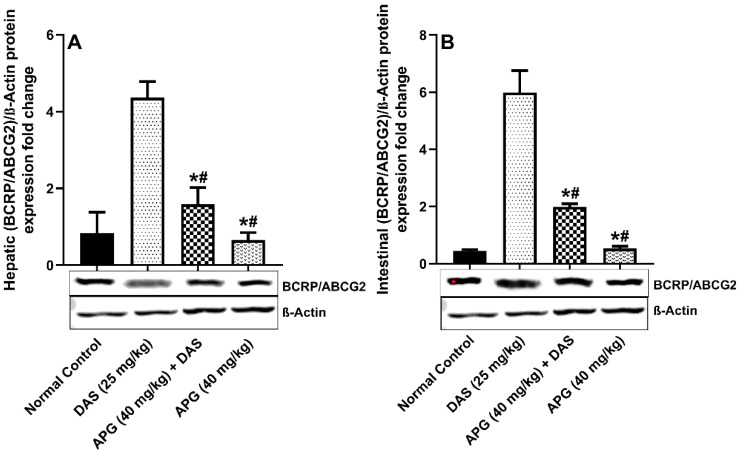
Hepatic (**A**) and intestinal (**B**) BCRP/ABCG2 protein expression in rats after DAS administration with or without APG pretreatment. All results are presented as the average ± SD. * *p* < 0.05 (Normal control); # *p* < 0.05 (DAS).

The inhibitory potential of APG on BCRP/ABCG2 protein expression was investigated and shown in Figure 5A,B. hepatic and intestinal BCRP/ABCG2 protein expression was significantly reduced after APG pretreatment compared to that in normal rats. DAS peroral administration caused significant induction of 92.55% and 95.66%, respectively, (*p* < 0.05) hepatic and intestinal protein expression of BCPR/ABCG2 compared to that in normal rats. The APG-pretreated rats showed significant inhibition of the BCPR/ABCG2 protein that is (66.77% and 41.06% inhibition, respectively) as compared to DAS only (*p* < 0.05). APG (40 mg/kg) only pretreatment led to the inhibition of 91.05% and 91.50% of hepatic and intestinal BCRP/ABCG2 protein expression as compared to normal rats.

## 3. Discussion

Chemotherapeutic drugs are extremely toxic and come with a wide variety of severe side effects. Cancer patients often use complementary therapies, such as herbal supplements, to lessen the unpleasant side effects of chemotherapy and improve their overall health [24]. The probable risk from HDI makes the benefits of using such herbs unwarranted. HDIs is undesirable in the management of cancer because of their swift dose effect relationship. Inhibition and/or induction of drug-metabolizing enzymes (DME) and or drug transporters mediated by herbs is the most typical mechanism of herb-drug interactions, which in turn alters the pharmacokinetic disposition of the drug [24,25]. Chronic myelogenous leukemia (CML) and Philadelphia chromosome-positive acute lymphocytic leukemia patients who did not respond to first-line therapy are two of the cancer types for which DAS has been approved. BCR-ABL and Src family kinases are both inhibited by DAS, a new tyrosine kinase multitarget inhibitor [5]. DAS is broadly metabolized in the liver and intestine by the CYP3A4 [7]. In addition, DAS is capable of induction, and is vulnerable to pharmacokinetic interaction. Drug–herb interaction (DHI) risks are possible with concomitant usage of APG along with DAS [8,9,10]. The purpose of the current study was to determine whether or not APG has a pharmacokinetic interaction with DAS, and if so, how they would interact. The current study aims to study the probable interaction mechanism between APG pretreatment and DAS using pharmacokinetic analysis. The UPLC MS/MS technique was used to examine DAS levels in rat plasma. The sensitivity and specificity of the analytes were enhanced by adjusting the mass spectrometer settings. Precision and accuracy were validated according to the “US Food and Drug Administration 2018 guideline for bioanalytical method validation”. In both intraday and interday samples, differences in precision and accuracy were found to be well within the ±15% range which is considered acceptable.

The concomitant use of supplements together with prescription medications, particularly those with a narrow therapeutic range, may lead to drug interactions. The wide spread use of herbs/herbal supplements/herbal medications with an estimated 40 to 60% of the US population with chronic disease and 20–25% patients with prescribed medication procuring herbal supplements [26,27]. DAS has already been linked to a potentially harmful herb/drug interaction [11,28,29,30]. There is minimal risk associated with APG’s long history of safe use in food, herbs, and herbal supplements. Pharmacokinetic interaction between herbs and pharmaceuticals is possible due to APG, a strong inhibitor of the CYPP450 enzyme system and the drug transporters Pgp and BCRP [19,20,21,22]. Therefore, APG has ability to change the pharmacokinetic profile of DAS absorption, distribution, metabolism, and elimination (ADME) via the regulatory actions of the efflux pumps PGP and BCPR and cytochrome P450 enzymes. The probable pharmacokinetic interactions may take place with APG as a result, it is necessary to explore the safety concerns associated with such simultaneous or parallel usage. The radical alteration in the pharmacokinetic parameters of DAS 25 mg/kg P.O. were examined with and without APG pretreatment in rats. Research on rats that were pretreated with APG revealed an increase in the plasma concentration of DAS. The rapid rate of absorption, as shown by the sharp increases in pharmacokinetic parameters such as Cmax, AUC_(0-t)_, AUMC_(0-inf obs)_, T_1/2_, Tmax, and MRT after APG 40 mg/kg pretreatment in rats, may be attributable to the substantial suppression of CYP3A2, Pgp/MDR1, and BCPR/ABCG2-mediated DAS metabolism in the liver and intestine. The similar pharmacokinetic parameters were exhibited from DAS interaction with St. John’s wort, fruit juices, and moderate/strong CYP3A inhibitors have the ability to escalate Cmax, AUC_(0–t)_, MRT, T_1/2_ and suppress the rate of Vd, the rate of clearance Cl, and Kel constant, thereby increasing bioavailability [10,11,31,32,33]. The increase in systemic bioavailability in plasma might be attributed to substantial suppression of CYP3A2, Pgp/MDR1, and BCPR/ABCG2-mediated DAS metabolism in the liver and intestine of DAS due to APG [21,23,34,35,36,37].

DAS has been shown to increase the exposure of CYP3A4 substrates simvastatin and midazolam in humans and cyclosporine in rats in investigations [9,10,11]. The outcomes of an immunoblot experiment executed on liver and intestinal tissues in all groups to explore for CYP3 A2, Pgp-MDR1, and BCPR/ABCG2 protein expression. In rats co-administered DAS/APG and APG alone, substantially suppressed the elevated CYP3A2, Pgp-MDR1, and BCPR/ABCG2 protein expression in the liver and gut compared to DAS alone. APG is a bioactive polyphenol with the potential to influence CYP3A2/Pgp-MDR1/BCPR/ABCG2 in the liver and gut. APG pretreatment inhibits the expression of CYP3A2, Pgp-MDR1, and BCPR/ABCG2 proteins in the liver and gut of rats, according to our results. APG treatment substantially modulates the pharmacokinetics of DAS and pharmacokinetic parameters further validates these where Cmax), AUC_(0–t)_, AUMC_(0-inf_obs)_, T_1/2_, Tmax, and MRT and reduced the rate reduced the Vd, Kel, and Cl, thus escalating systemic bioavailability and rate of absorption that is mediated by CYP3 A2, PGP-MDR1, and BCPR/ABCG2. This present study further endorses the previous pharmacokinetic research that demonstrated interactions with CYP3A, Pgp/MDR1, and BCPR/ABCG2 inhibitors enhanced the DAS bioavailability [28].

APG has a minimal risk of harm to humans and has been used for centuries in food, herbs, health supplements, and traditional medicine. It can be used at the same time as other medications if necessary. DAS ADME pharmacokinetics may be influenced by APG due to the regulatory actions of efflux pumps Pgp and BCPR and cytochrome P450 enzymes CYP3A2. Consequentially, more systematic clinical study on herb–drug interactions is essential, as is proper regulation in herbal safety and efficacy, as well as the avoidance of potentially fatal medication interactions when APG-containing foods or traditional herbs are used alongside DAS.

## 4. Materials and Methods

### 4.1. Materials

Dasatinib (DAS), apigenin (APG), and ibrutinib (IBR), were obtained from Sigma-Aldrich (St. Louis, MI, USA). Acetonitrile, formic acid, methanol, and ammonium acetate were purchased from BDH, Pool (UK) and antibodies anti-P-gp/MDR1/ABCB1, anti-BCRP/ABCG2, Anti-CYP3 A2, and anti-β-actin antibodies were obtained from Santacruz (USA).

### 4.2. Animals and Pharmacokinetic Studies

The Central Animal House facility of College of Pharmacy, King Saud University’s, Riyadh, Saudi Arabia, provided 24 healthy adult male Wistar rats (weighing 211–223 g), and the experiment was authorized by the Animal Ethics Committee of the College of Pharmacy, King Saud University (KSU-SE-21-58). All animals were retained in polypropylene cages: 6 animals were caged (n = 6) per cage with a 12-h light/dark cycle, at 25 °C in humane conditions with free access to food and water and adapted to the living conditions one week prior to experiment

### 4.3. Experimental Design

The animals were categorized into four groups (n = 6) and fasted for 12 h before the experiment. Group I and II were orally administered with normal saline for 7 days and Group II was orally administered DAS (25 mg/kg) on the 7th day. Group III was co-administered DAS with APG for 7 days and DAS (25 mg/kg p.o.) on the 7th day, 2 h after APG 40 mg/kg administration, and Group IV was administered with APG 40 mg/kg for 7 days. The blood was collected from the tail vein at various time points after DAS administration and subjected to centrifugation at 5000× *g* for 10 min to obtain plasma for DAS quantification using ultra pressure liquid chromatography with mass. At the end of the experiment, all animals were euthanized using carbon dioxide, and liver and intestine tissues were harvested and for Western blotting.

### 4.4. Mass Spectrometry and UPLC Chromatographic Conditions

Using UHPLC-MS/MS, DAS concentrations in rat plasma samples were determined. A mixture of DAS and IBR (internal standard) was separated on an Acquity BEH C18 column, and both compounds were eluted at a flow rate of 0.25 mL/min from a mobile phase consisting of acetonitrile, 0.1% formic acid, and 20mM ammonium acetate (95:5). The entire duration of the study was about 2.5 min. The auto-sampler was kept at 10 °C, while the column oven was kept at a constant 40 °C. Electrospray ionization was used to ionize samples in a positive direction. In the concentration range examined (5–2500 ng/mL), the calibration curves displayed linearity over the concentration range. Multiple reaction monitoring (MRM) mode detection and quantitation of analyte (DAS) and IS (IBR) employed the precursor to product ion transition of 488.06 and gt; 401.1 and 441.16 and gt; 84.04, respectively. Capillary voltage was 3.4 kV, source temperature was 150 °C, and desolvation temperature was 350 °C for optimum sample ionization using MS/MS. A flow rate of 600 L/h of nitrogen gas was utilized for the desolvation process, whereas a flow rate of 0.18 mL/min of argon gas was used for the collision process. The analyte and IS cone voltages were 46 V and 48 V, and the collision energy was 28 eV and 40 eV, respectively. Precision and accuracy were partially confirmed for the assay using the “US Food and Drug Administration 2018 guideline for bioanalytical technique validation,” and found to be within acceptable ranges of 15% both intra- and inter-day. The samples were acquired using Masslynx 4.1 SCN 805, and then processed using TargetLynks. After making a few modifications to a previously reported study, it was put to use [38]. 

### 4.5. Sample Preparation

A plasma sample of 150 µL was aliquoted into an Eppendorf tube, along with 20 µL of a spiked internal standard (250 ng/mL), and 1 mL of ethyl acetate was also added for the extraction phase. For 10 min at 4 °C and 10,500 rpm, centrifuged samples were shaken for 15 min after being vortexed. Finally, 800 µL of the organic upper layer were transferred to an Eppendorf tube and dried in a sample concentrator at room temperature. Following reconstitution with 150 µL of acetonitrile, the dried residue was transported to UHPLC-MS/MS for 5 µL of analysis.

### 4.6. Pharmacokinetic Analysis

PK Solver software was employed to calculate the pharmacokinetic parameters using non-compartmental model. The calculated parameters were as follows: half-life (T_1/2_), elimination rate constant (Kel), time to maximum concentration (Tmax), the maximum concentration (Cmax), area under concentration time curve (AUC), the area under the moment curve (AUMC); the volume of distribution (Vd), the mean residence time (MRT), and clearance (Cl) were examined.

### 4.7. Protein Expression Analysis

A PierceTM BCA Protein Assay Kit (Thermo Fisher Scientific) was used to extract the total protein from the hepatic and intestinal tissues by bicinchoninic acid technique [39]. Immunoblots were executed as per the methods of Towbin et al. (1979) with slight modification [40,41,42]. Briefly, 10% SDS polyacrylamide gels were used to electrophorese 25 μg of hepatic and intestinal epithelial proteins. These proteins were then transferred to activated PVDF membranes and blocked with 4% skim milk and BSA in TBST. After that, membranes were treated with antibodies for CYP3A2 isozymes, active transporters P-glycoprotein/MDR1, BCRP/ABCG2, and β-actin protein expression for an overnight period (4 °C). The membrane strips were incubated with the suitable secondary antibodies for 2 h after repeated washing steps with 1% TBST and TBS (room temperature). The membrane strips were then washed four times with TBST for five minutes each time. Bands were developed using Luminata™ Western Chemiluminescent HRP Substrates (Millipore), followed by densitometric evaluation of the immunoblots.

### 4.8. Statistical Analysis

The data were presented as a mean SE (SEM). Significance (*p* < 0.05) was determined using one-way analysis of variance followed by Dunnett’s post hoc test (against respective controls).

## 5. Conclusions

Using the UPLC-MS/MS technique, we effectively analyzed the pharmacokinetic interaction between APG and DAS in rat plasma after single and co-oral dosing. APG pretreatment has the potential to radically alter the pharmacokinetic parameters of DAS, increasing systemic bioavailability and absorption rate, as shown by in vivo pharmacokinetics and protein expression of cytochrome P450 3A2, peptidyl guanylate cyclase MDR1, and phosphodiesterase type 5 inhibitor 2. Consequentially, more systematic clinical study on herb–drug interactions is recommended, as is proper regulation in herbal safety and efficacy, as well as the avoidance of potentially fatal medication interactions when APG-containing foods or traditional herbs are used alongside DAS.

## 6. Limitations and Future Prospects

Based on the findings of the present investigation, it was hypothesized that APG would influence the pharmacokinetics of CYP3A/Pgp substrate drugs in humans. However, quantitatively extrapolating these results to humans is challenging because the biotransformation rate of DAS in rats varies from that in humans. This study’s findings, however, were derived solely from studies conducted with rats. Further thorough in vivo studies of the APG–DAS interaction in humans are warranted to support these findings. Effective care requires health care providers to have an open discussion with patients about their use of herbal medications and to take careful notes. The possible benefits and risks of herbal products in cancer treatment should be made clear to patients. Patients should be aware of the risks of HDIs, including increased toxicity or ineffective treatment. Additionally, DAS drug monitoring is recommended when adverse events, such as toxicity or lack of clinical response, are suspected. Also, it is crucial to record any adverse events and share them with the pharmacovigilance network is of utmost importance.

## Figures and Tables

**Table 1:** Pharmacokinetic parameters (non-compartmental) of dasatinib along with apigenin following an oral administration in rats.

Parameter (Unit)	DAS 25 mg/kg ± SD	DAS + APG ± SD	% Change
K*el* (1/h)	0.14 ± 0.01	0.11 ± 0.004 *	24.83
T_1/2_ (h)	4.97 ± 0.48	6.56 ± 0.26 *	32.16
Tmax (h)	1 ± 0	1.5 ± 0.00 *	50
Cmax (ng/mL)	263.06 ± 12.33	511.81 ± 39.50 *	94.56
AUC_0-t_ (ng/mL × h)	1635.84 ± 45.29	2947.80 ± 168.32 *	80.20
AUC_0-inf_obs (_ng/mL × h)	2063.49 ± 96.99	4129.07 ± 361.71 *	100.10
AUMC_0-inf_obs_ (ng/mL × h^2^)	16200.10 ± 1656.57	39472.75 ± 3665.28 *	143.65
MRT 0-inf_obs (h)	7.84 ± 0.46	9.55 ± 0.43 *	21.90
Vd (mg/kg)/(ng/mL)	0.087 ± 0.005	0.06 ± 0.004 *	33.69
Cl (mg/kg)/(ng/mL)/h	0.012 ± 0.0005	0.006 ± 0.00 *	49.95

Results are presented as the average ± SD. * *p* < 0.05 (DAS).

## Data Availability

All the data generated from the study are clearly presented in the manuscript.
